# Patient and public involvement mobile workshops – convenient involvement for the un-usual suspects

**DOI:** 10.1186/s40900-018-0123-1

**Published:** 2018-10-24

**Authors:** Abi Eccles, Carol Bryce, Amadea Turk, Helen Atherton

**Affiliations:** Warwick Medical School, University of Warwick, Coventry, CV4 7AL UK

**Keywords:** Family practice, General practice, Patients, Research design, Parents, Volunteers

## Abstract

**Plain English summary:**

When planning a research project into patients’ experiences of online booking of GP appointments, we tried out a new way to get feedback from the public on our research ideas and design. As the research topic is about GP services used by the general public, we wanted to get feedback from people with a broad range of backgrounds and perspectives. However, relying on individuals to firstly want to volunteer and then to take time to travel to and attend such an event, means that involvement may only be attractive to certain people. Others less interested in being involved – or those with busy schedules and additional responsibilities – may be unlikely or unable to attend.

With this in mind, we ran a series of mobile workshops designed to be particularly convenient to attend. Each workshop was arranged at a time and a place where potential volunteers were already present and available. For example, at a workplace or a social group during a scheduled break or popular time. This meant each workshop was convenient to attend as they were at a suitable time with no travel. They also were short, lasting 30 min, to minimise disruption to individuals’ diaries. To make taking part appealing, attendees were also paid (which is standard practice for patient and public involvement). This paper summarises and evaluates the process of running these workshops.

**Abstract:**

**Background**

Patient and public involvement in research is a quickly-evolving area, with investigators developing new approaches in recent years. One concern about patient and public involvement is that it only appeals to certain individuals. When designing research into online GP services – a topic relevant to the general population – we recognised the importance of involving members of the public with a broad range of backgrounds who may not have the time, resources and inclination to volunteer normally.

**Methods**

We devised a strategy that aimed to involve members of the public from varied backgrounds, who would not typically be able to be involved. We ran a series of one-off mobile workshops at existing organisations where potential volunteers were already in situ. The workshops were kept short, making them convenient and easily accessible. Volunteers were also paid, to ensure taking part was appealing.

**Results**

We ran a series of 4 workshops involving 26 members of the public with office workers, supermarket staff, gym members (and their friends) and parents attending a toddler group. Overall the workshops were successful, as they enabled us to gain varied perspectives from volunteers with a broad range of backgrounds, many of whom had not previously been involved in research. A key challenge was making initial contact with members of approached organisations. This indicates that it may be beneficial to consider how to make the workshops appealing, not just on an individual level, but at an organisational level too. A carefully planned design worked as it enabled large amounts of input in a limited amount of time, apart from one workshop (the parent group) due to practical reasons. This highlighted some limitations of this approach that could be addressed by adapting the workshop design, according to the organisation with which they are being run.

**Conclusion**

Running one-off mobile workshops at already existing organisations allowed us to involve members of the public from a broad range of backgrounds, who would not typically volunteer to be involved in research. This was particularly suitable as the topic we were designing research for – booking GP appointments – is relevant to the general public.

## Background

In recent years, the importance of patient and public involvement (PPI) in research processes has become increasingly recognised, with ever-evolving ways to involve members of the public and patients in research design, processes and dissemination. Most funding bodies now require a PPI plan, although this is to varying degrees. Reaction to PPI from researchers and academics appears to be mixed, with some quick to recognise the potential value of PPI, whereas others have been more sceptical about its worth [1].

If aiming to develop a PPI group membership that represents views of the wider population, a subsequent issue arises in how representativeness is defined. Notions of ‘representativeness’ can be contentious and have been previously examined within the PPI literature, often in reference to decisions about healthcare delivery and policy development [2, 3] as well as research processes [4]. One of the concerns of PPI is that members of the public who volunteer to take part are often from specific backgrounds where involvement is more accessible to them, e.g. they may have previous PPI experience or already be connected to the organisation carrying out the research. Some have suggested that organisers targeting invitations at certain individuals, alongside the self-selection by volunteers themselves, inevitably leads to sub-groups of the population being better represented than others [3]. Barriers to involvement for ‘ordinary’ people have also been identified, including time constraints, prioritisation of other activities and commitments, lack of expertise [2], incapacity to accommodate out-of-pocket expenses (whilst awaiting reimbursement), lack of transport and inconvenience of travel [4]. A tension also exists between aiming to involve ‘ordinary’ people, whilst also requiring a level of skill and investment beyond what the average person may be able to provide [2].

Recruiting PPI group members that reflect the wider population they aim to represent can be problematic [5] and as definitions or criteria for representativeness varies, then legitimacy of representation is easily challenged [4]. This problem is further compounded when involving members of the public in research about delivery of general practice. Typically, organisers invite individuals to volunteer for PPI due to specific groups they belong to relevant to the research question e.g. disease-specific or age-specific research [6]. General practice service provision however, concerns all members of public, with 90% of NHS appointments being in primary care [7], thus deciding whom to invite to take part without omitting certain social groups is challenging.

General practice is a service provided for the general population, therefore a PPI group for general practice research would ideally reflect a broad range of demographics. Patient Participation Groups in general practice are long established and exist in over two thirds of practices [8]. These are a form of PPI but are known to face recruitment difficulties particularly in relation to age and ethnicity [8]. Broadening the reach beyond existing groups requires reaching out to those who do not necessarily identify as ‘patients’ and have less inclination and/or resources to take part in PPI activities. It is widely accepted that consulting a wholly representative group is beyond the remit and aims of PPI [4], however seeking breadth in identifying those who might be affected by the issue at some point may be more attainable. For research conducted in general practice settings, appropriate representation may be conceptualised in various ways. We may aim to reflect the demographic make-up of the wider population, or to include a broad range of socioeconomic backgrounds as this is a major determinant of health status [9], or it could be based upon a broad range of health conditions (including absence of), or a range of healthcare service use, or differing ideologies.

Those less likely to volunteer for research activities (involvement and participation) are often described as ‘hard-to-reach’ populations. This term typically refers to specific marginalised or vulnerable groups within societies (e.g. victims of domestic abuse, homeless, people with learning disabilities or ethnic minorities) but the term has been criticised as it focuses on how public members’ characteristics mean they are less likely to be involved, and overlooks how PPI strategies may also shape access to involvement [10]. Another challenge also lies in involving members of the public who are not necessarily marginalised or vulnerable, but due to their circumstances (e.g. time pressures or other commitments) are ‘hard-to-involve’. In similar ways that organisers may not be providing suitable opportunities for so called ‘hard-to-reach’ groups (5, 10), they may also better design PPI activities to make them more accessible for those ‘hard-to-involve’. We aimed to overcome this challenge by piloting a series of mobile PPI workshops that were designed to be particularly convenient and accessible for those who would not normally have opportunities, resources, time or inclination to be involved. For example, people working full-time, those unable to travel, those with childcare commitments and who would not normally go out of their way to attend PPI events.

## Methods

We designed, piloted and evaluated a series of four mobile PPI workshops, with aims to involve members of the public with a broad range of backgrounds, who would not typically volunteer for PPI. The broader aims of the workshops were to gain insights from attendees to design research into online booking of GP appointments, in preparation for a grant application.

### Workshop design

**Fig. 1:**
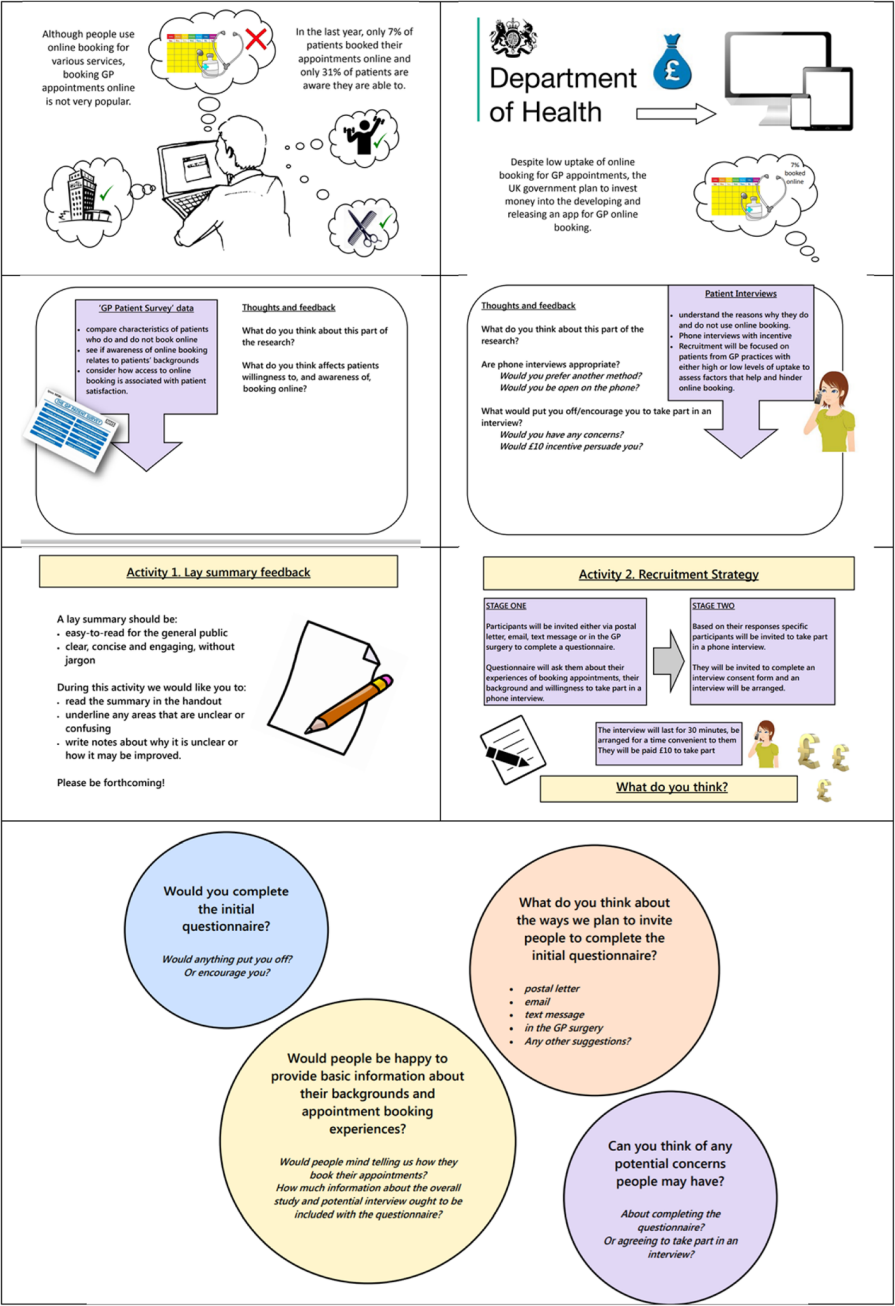
Infographics used across the workshops

The workshops were designed to be run with up to 10 members of the public. Each workshop followed a schedule (see Table 1) and lasted no more than 30 min to minimise disruption to attendees’ diaries. They were arranged at already-existing organisations to allow convenient attendance and attendees were provided with refreshments and received payment as an incentive to take part. Content differed with each subsequent workshop, dependent on research proposal development. Tools such as infographics to convey information clearly (see Fig. 1. Infographics used across the workshops), post-it notes for recording ideas to facilitate discussion and worksheets were used within the workshops to maximise input from attendees within a limited timescale.

**Table 1:** Workshop schedule

*Duration: 30 min*	
0–10 min – introduction	
• Welcome and thank attendees for joining us.	
• Introduce ourselves, purpose of PPI and aims of workshop.	
• Present infographics.	
10–20 min – discussion in small groups	
• Put attendees into small groups to discuss key points.	
◦ Attendees use post-it notes to record their perspectives.	
◦ These are added to large infographics in front of them.	
◦Ask attendees to be forthcoming with their perspectives.	
◦ *If you think of something you don’t think is important, please include it. If you think something may be controversial, please include it. This is the point of the workshops, it’s to gain a broad range or perspectives and identify areas we have overlooked.*	
20–30 min – group discussion and summary	
• Have a group discussion about each of the areas using post-it notes to guide content.	
• Summarise the issues in bullet points.	
• Ask if they feel anything is missing.	
Post-workshop	
• Thank everyone for their time and input.	
• Distribute the claim forms, demographic forms and the contact forms.	
• Explain: *They will be paid £10* via *bank transfer and there is a claim form for them to complete. They can leave their contact details if they wish to receive a summary of workshops directly or if they wish to be kept informed about any future opportunities. If they have any further input, our contact details are in the leaflet. There is a form asking you complete background information. This will be kept completely anonymous and will be used by to ensure we have a broad range of people in workshops.*

### Workshop organisation

We initially contacted various organisations via email, letter or phone. Contact details were obtained from organisations’ website or via a known contact. Once contact was made with a willing individual from an organisation, we scheduled a workshop and the contact invited members of the organisation to attend. Two researchers attended each workshop, one to run the workshop and the other to take notes.

We gave all PPI attendees the opportunity to be involved with future PPI events after the workshop (should the proposed research be awarded funding) and/or to be sent a summary report for the workshops once they were complete. However, each workshop included a different group of people and were run as one-off events. We felt a changing pool of individuals was reflective of a primary care population and would encompass a broader range of perspectives. Such an approach may seem to disregard a key component of PPI, which is to build long term reciprocal relationships with PPI members. Considering this, it was made clear to PPI attendees what they could expect from taking part via an information leaflet. Those who registered their interest may be invited for future involvement, providing opportunities to build longer term reciprocal relationships.

As these workshops were to inform the design of research into online booking of GP appointments, we wanted to involve people from a broad range of demographic backgrounds. The time and setting of the workshops varied, reflecting how we as organisers purposely arranged meetings to be convenient for attendees. We ran four workshops with 1) office workers during a lunch break in their place of work, 2) parents at a drop-in toddler group during a weekday, 3) supermarket staff before a shift started at their place of work, and 4) leisure centre gym members and acquaintances at a neighbouring community centre during a weekday evening. We also asked attendees to provide anonymised information about their backgrounds, health conditions and previous involvement in research to assess the breadth of backgrounds of those who attended.

## Results

### Background of attendees

We involved 26 members of the public in a series of four workshops (5–8 participants) between May and October 2017. A key aim of this strategy was to involve people who would not typically volunteer to be involved in research from a broad range of backgrounds. The ages of those who volunteered ranged from 21 to 68 years, the average being 40 years old. More females than males volunteered (65%, 17/26), but it is worth noting that attendees at that parent and toddler group were all female and without this group, proportions are more equal (43% male and 57% female). Seven out of the 26 volunteers (27%) reported having a long term condition, a figure close to the proportion of those with long term conditions in England (30%) [11]. On the whole volunteers lacked ethnic diversity, as the majority 24/26 described themselves as white and/or British, one as Turkish and one as European. There was a broad range of volunteers in terms of their employment, these are categorised in Table 2. Only a minority (3/19 of those asked) reported that they have previously been involved in research, although we do not know whether this was specifically PPI or as research participants.

**Table 2:** Broad employment categories of attendees

Administration	
Company director	
Design and engineering	
Teacher	
Retail and customer services	
Sustainability champion	
Warehouse assistant	
Baker	
Pharmacist	
General assistant	
Unemployed	
Medical writer	
Receptionist	
Health and wellbeing	
Project coordinator	

### Barriers and facilitators to conducting the workshops

Encouraging organisations to allow us to run a workshop in the first instance was often challenging and initial invitations typically were not responded to. Although there were incentives to individuals to take part in the workshops, those in positions to authorise such workshops seemed unwilling or did not respond to initial contact. For example, we contacted several local supermarkets, warehouse distribution centres, factories, public services (fire, police and school) without success. For some the timing was problematic as it happened to be a busy time of year, for some their schedules were too unpredictable to accommodate a workshop and others were unable to gain authorisation at the company level. In the majority of cases we have no record of reasons behind such reluctance, but we assumed this could be because accommodating a workshop in their organisation’s schedule may have risked disruption without incentives for the organisation itself. Methods to overcome this barrier are worth further consideration in planning such workshops.

The success of organising the workshops typically depended on contacting an individual from within an organisation who was reliable, organised and interested in taking part. For example, we contacted an individual who worked as a ‘Community Champion’ for a local supermarket who was integral to that workshop’s success. It is noteworthy that we had attempted contact with others in this role from other supermarkets without success, illustrating the importance of an engaged and willing individual within the organisation. Arranging a workshop relied on the contact to organise a suitable meeting room and to invite members within the organisation. Such contacts emerged by chance (as the contact responded to initial invite) or through research team members who have already-established relationships with a member from the organisation. Two of the four workshops were arranged through established contacts. We invited two other established contacts to help us arrange a workshop but this was not possible as the workplaces in question would not permit their staff to participate during working hours. .

Overall, the workshops went as planned and according to carefully designed schedules. However, following the schedule with parents from a toddler group was not possible due to interruptions and distractions from the children. This brought attention to the importance of adapting the workshop design depending on the setting and attendees. For example, it may have been more conducive to have set up a drop-in stand in this setting, to consult individuals or pairs at a time convenient to them.

### Workshops’ outcomes

Involvement from attendees during these workshops was fed back to the research team and guided the development of our research proposal. We confirmed that the research topic – online booking of GP appointments – was an area relevant to members of the public. Through the workshop discussion we were able to provide additional detail to our argument, justifying reasons why it was an important area to research from patients’ perspectives. The workshops also aided the selection of methods that were convenient and appealing to potential research participants.

We found that opinions and concerns regarding the topic and research design differed between the workshops, allowing us to consider a broad range of perspectives in the study design. A lay summary for the grant application was also scrutinised by attendees using a worksheet and group discussion. In doing so attendees identified inaccessible language and content deemed less relevant, that the research team had not previously recognised as problematic.

### Future involvement

Although the workshops were set up for one-off consultation with members of the public, 11 out of the 26 attendees registered interest in future involvement. This further supported our judgement that the mobile workshop format enabled us to reach those who are not typically inclined to volunteer for PPI, whilst also providing potential opportunities to develop longer term reciprocal relationships with those who opted for continued involvement more akin to traditional PPI strategies. If the research grant is awarded, we plan to invite those interested, to take part in future PPI activities. The process of carrying out the workshops also allowed us to build rapport with one particularly interested attendee who subsequently became named as a co-applicant on the grant application. He will act as a key member of the research team in his role of PPI representative, if the grant is awarded.

## Discussion

Without wishing to devalue the contributions of those who do give up time and go out of their way to volunteer for PPI opportunities, we deemed gaining insights from those less likely to volunteer as valuable. This issue is pertinent in the context of general practice research, which provides services for people with a broad range of backgrounds, attitudes and responsibilities.

Some may argue that aiming for representativeness in the context of PPI is less important than other attributes PPI groups may possess. For example, disease-specific research may particularly benefit from input of individuals with much experience in a specific condition or later stages of research (such as data collection or dissemination) may require input from those experienced and familiar with a research environment and PPI processes. However, as all members of the public are likely to use GP services, PPI that represented a broad range of perspectives was preferable in the context of primary care research. The PPI was also to inform the early stage of research design, so specific skills or experience in research or PPI were not necessary. We envisage more specific and experienced PPI will be appropriate as the project progresses (if funding is awarded).

PPI databases and networks, general advertising, charities, and support groups are valuable approaches to finding volunteers, especially when targeting specific patient populations. However, such approaches may only appeal to ‘the usual suspects’, i.e. those with the inclination, time and resources to volunteer. Although we would not suggest those 26 who volunteered in our workshops were representative of the general population, we were satisfied we had involved people from a broad range of backgrounds who would not typically be reached by usual PPI strategies. Using this format –mobile workshops of limited duration in varied settings – appears to help broaden PPI involvement from a diverse group of people.

As mentioned previously, the one-off nature of the workshops may seem to disregard the value of long term reciprocal relationships with PPI volunteers. However, it is important to note that these workshops were one facet of a more comprehensive evolving PPI strategy. Once funding for further research is secured, the volunteers who expressed interest will be invited to take part in future involvement. Furthermore, our co-applicant will have a key role as PPI representative throughout the research, similar to other research projects where PPI representatives have played integral roles [12].

## Conclusions

Mobile workshops provided a novel strategy for PPI which – to our knowledge – has not been used previously. Running mobile PPI workshops at existing organisations and keeping them short, allowed us to make it convenient, accessible and appealing to potential volunteers. We demonstrated the workshops were successful in involving members of the public with a broad range of backgrounds, the majority of whom had not previously volunteered for PPI. We highlighted some of the challenges faced when organising and running workshops (e.g. making initial contact, gaining permission and group dynamics) and suggested ways to overcome these (e.g. an engaged individual within organisation, incentives at an organisational level and adapting workshop design).

## Abbreviation

PPI: Patient and public involvement
